# Therapeutic Potential and Mechanisms of Rosmarinic Acid and the Extracts of Lamiaceae Plants for the Treatment of Fibrosis of Various Organs

**DOI:** 10.3390/antiox13020146

**Published:** 2024-01-24

**Authors:** Yong Chool Boo

**Affiliations:** 1 Department of Molecular Medicine, School of Medicine, Kyungpook National University, 680 Gukchaebosang-ro, Jung-gu, Daegu 41944, Republic of Korea; ycboo@knu.ac.kr; 2 BK21 Plus KNU Biomedical Convergence Program, Department of Biomedical Science, The Graduate School, Kyungpook National University, 680 Gukchaebosang-ro, Jung-gu, Daegu 41944, Republic of Korea; 3 Cell and Matrix Research Institute, Kyungpook National University, 680 Gukchaebosang-ro, Jung-gu, Daegu 41944, Republic of Korea

**Keywords:** rosmarinic acid, fibrosis, Lamiaceae, transforming growth factor β1, TGF-β1, Wnt, peroxisomal proliferator-activated receptor γ, PPARγ, 5′ AMP-activated protein kinase, AMPK, erythroid 2-related factor 2, NRF2

## Abstract

Fibrosis, which causes structural hardening and functional degeneration in various organs, is characterized by the excessive production and accumulation of connective tissue containing collagen, alpha-smooth muscle actin (α-SMA), etc. In traditional medicine, extracts of medicinal plants or herbal prescriptions have been used to treat various fibrotic diseases. The purpose of this narrative review is to discuss the antifibrotic effects of rosmarinic acid (RA) and plant extracts that contain RA, as observed in various experimental models. RA, as well as the extracts of *Glechoma hederacea*, *Melissa officinalis*, *Elsholtzia ciliata*, *Lycopus lucidus*, *Ocimum basilicum*, *Prunella vulgaris*, *Salvia rosmarinus* (*Rosmarinus officinalis*), *Salvia miltiorrhiza*, and *Perilla frutescens*, have been shown to attenuate fibrosis of the liver, kidneys, heart, lungs, and abdomen in experimental animal models. Their antifibrotic effects were associated with the attenuation of oxidative stress, inflammation, cell activation, epithelial–mesenchymal transition, and fibrogenic gene expression. RA treatment activated peroxisomal proliferator-activated receptor gamma (PPARγ), 5′ AMP-activated protein kinase (AMPK), and nuclear factor erythroid 2-related factor 2 (NRF2) while suppressing the transforming growth factor beta (TGF-β) and Wnt signaling pathways. Interestingly, most plants that are reported to contain RA and exhibit antifibrotic activity belong to the family Lamiaceae. This suggests that RA is an active ingredient for the antifibrotic effect of Lamiaceae plants and that these plants are a useful source of RA. In conclusion, accumulating scientific evidence supports the effectiveness of RA and Lamiaceae plant extracts in alleviating fibrosis and maintaining the structural architecture and normal functions of various organs under pathological conditions.

## 1. Introduction

**Figure 1:**
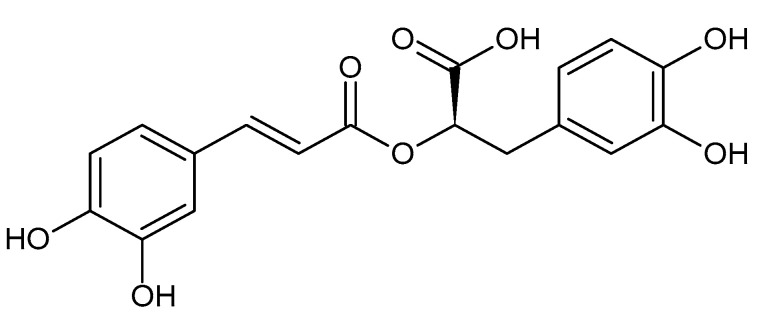
The chemical structure of rosmarinic acid (RA).

In 2007, the mechanism of action of Wen-pi-tang-Hab-Wu-ling-san (WHW), which is an oriental herbal prescription currently used in Korea for the treatment of chronic renal failure, was investigated in our laboratory [1]. WHW extract inhibited the expression of alpha-smooth muscle actin (α-SMA) and the phosphorylation of small mothers against decapentaplegic (SMAD) 2 that were induced by transforming growth factor (TGF) β1 in Madin–Darby canine kidney cells (MDCKs), thus supporting its renal protective effects via the inhibition of the TGF-β1/SMAD2 pathway. In the same study, rosmarinic acid (RA, Figure 1) was proposed as an active compound responsible for the antifibrotic activity of WHW extract. At that time, there was little research regarding the antifibrotic actions of RA; however, since then, related research has substantially increased.

Fibrosis, which causes structural hardening and functional degeneration of major organs of the body, is characterized by the excessive production and accumulation of connective tissues containing collagen and α-SMA [2,3]. Fibrosis is caused by chronic exposure to physical, chemical, and biological stresses, and its progression is mediated by various factors, such as TGF-β1, platelet-derived growth factor (PDGF), connective tissue growth factor (CTGF), Wnt, heat shock proteins (HSPs), and reactive oxygen species (ROS) [4,5]. Because the disease mechanism of fibrosis for each organ has both common and different elements, research has been conducted to identify the therapeutic targets specific to individual organs as well as treatment strategies to recover them to a normal state [6,7,8,9,10].

In traditional medicine in the East and West, herbal prescriptions that are composed of several medicinal plants have been used to alleviate fibrotic diseases of the major organs, including the liver, kidneys, and lungs [11,12,13]. Interestingly, plant extracts with these effects contain several polyphenolic compounds in common, and studies on the pharmacological effects of these compounds are attracting attention [14,15,16]. A literature search by Alberti showed that plant extracts that contain hydroxycinnamic acids inhibited experimentally induced liver fibrosis [17]. Contents of hydroxycinnamic acids, such as caffeic acid, chlorogenic acid, and RA, were particularly high in Lamiaceae plants, and RA was found to be the most common main compound in these plants. The antioxidant and anti-inflammatory properties of RA underlie its therapeutic potential against various liver diseases, such as hepatitis, fibrosis, cirrhosis, and carcinoma [18].

RA is a secondary metabolite that is found in various plants, including the Lamiaceae, Boraginaceae, and Apiaceae families, and it is structurally an ester compound of caffeic acid and 3,4-dihydroxyphenyllactic acid [19]. As a polyphenol compound, RA is considered to function as a defensive phytoalexin in plants and has multiple biological activities in addition to its antioxidant and antimicrobial properties [20]. Because oxidative stress, which is increased by various internal and external factors, leads to excessive inflammatory responses and various chronic diseases, the medical application of RA for its antioxidant and anti-inflammatory properties is currently underway [21,22]. The value of RA as a nutraceutical, which promotes the overall health of the body, is high, and the medical application of RA is gradually and continuously expanding, as discussed in recent review papers [23,24,25]. 

Compared to previous studies, this review was mainly focused on discussing the antifibrotic effects of RA and plant extracts that contain RA, as observed in various experimental models for fibrosis of the liver, kidneys, heart, lungs, and other organs, as well as evaluating their therapeutic potential for the treatment of human diseases.

## 2. Common Mediators of Fibrosis

**Figure 2:**
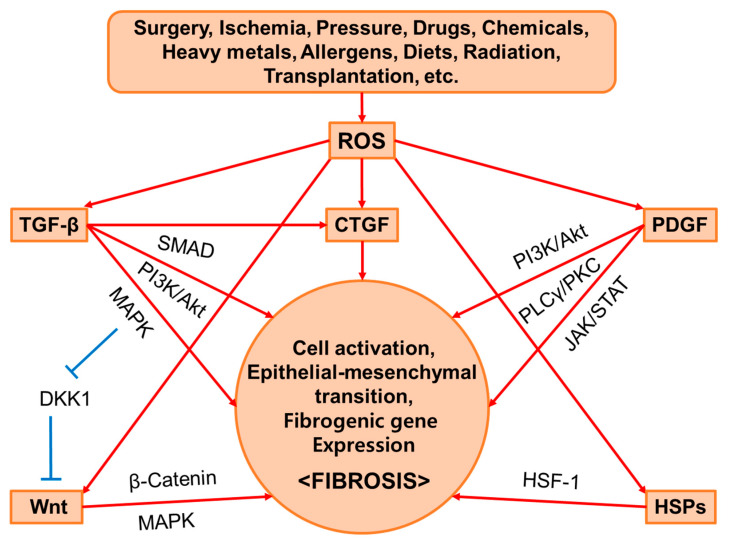
Common mediators of fibrosis. Surgery, ischemia, pressure, drugs, chemicals, heavy metals, allergens, diets, radiation, transplantation, etc. stimulate the production of intracellular reactive oxygen species (ROS) and cause oxidative damage, structural deformation, and functional abnormalities in various organs. These stresses stimulate the expression of transforming growth factor beta (TGF-β), platelet-derived growth factor (PDGF), Wnt, heat shock proteins (HSPs), etc. The canonical TGF-β signaling pathway is mediated by the small mothers against decapentaplegic (SMAD) and induces the expression of the target genes. As a result, the expression of connective tissue growth factor (CTGF) is induced and the signaling pathway that it mediates is activated. Additionally, the noncanonical TGF-β signaling pathway activates the Wnt signaling pathway by suppressing Dickkopf-1 (DKK1) through an extracellular signal-regulated kinase (ERK)-dependent mechanism. The activation of the canonical Wnt signaling pathway activates β-catenin and induces the expression of the target genes. The TGF-β, CTGF, and Wnt signaling pathways cooperatively induce cell activation, epithelial–mesenchymal transition (EMT), and the expression of fibrogenic genes, such as alpha-smooth muscle actin (α-SMA) and collagens to promote fibrogenesis. Sharp red arrows (→) indicate stimulation, and blunt blue arrows (⊥) indicate suppression.

In this section, we briefly provide background information on the main mediators of fibrosis. The regulatory roles of TGF-β, PDGF, CTGF, Wnt, HSPs, and ROS in cell activation, epithelial–mesenchymal transition (EMT), or target gene expression related to fibrosis are discussed. Simplified illustrations are presented in Figure 2. There may be additional factors that play a vital role in fibrosis even if they are not covered here.

### 2.1. TGF-β

The mammalian genome encodes TGF-β1, -β2, and -β3, which bind to the TGF-β receptor (TGF-βR) type I and type II transmembrane protein kinases [26]. TGF-β synthesis is triggered by tissue injury and mediates the activation of various cells that are involved in immunological responses and tissue remodeling [5]. In the canonical pathway, the binding of TGF-β to the membrane receptors stimulates the phosphorylation of SMAD2 and SMAD3, which, in cooperation with SMAD4, induces the transcription of the target genes involved in cell proliferation and transdifferentiation, extracellular matrix (ECM) synthesis, and the expression of tissue inhibitor of metalloproteinase (TIMP) and SMAD7 (a feedback inhibitor of TGF-β signaling) [27]. In the noncanonical pathways, TGF-β stimulates the mitogen-activated protein kinase (MAPK) signaling pathway and the phosphoinositide 3-kinase (PI3K)/protein kinase B (PKB, Akt)/mammalian target of rapamycin (mTOR) signaling pathway, which results in the transcription of target genes including CTGF, α-SMA, collagen, fibronectin, and other ECM components [26].

### 2.2. PDGF

The PDGF family members, including PDGF-AA, -AB, -BB, -CC, and -DD, bind to the tyrosine kinase PDGF receptor (PDGFR) dimers αα, αβ, or ββ to stimulate auto-phosphorylation and the activation of the cytoplasmic signaling pathways [28]. The binding of the PDGF isoforms to the relevant PDGFRs initiates distinct receptor dimerization and phosphorylation and provides multiple binding sites for several adaptor molecules, thereby activating various downstream signaling pathways mediated by PI3K, Akt, extracellular signal-regulated kinase (ERK), phospholipase C (PLC) γ, diacylglycerol/protein kinase C (PKC), Janus kinase (JAK), signal transducers and activators of transcription (STAT), and/or Notch [29]. PDGF-BB has been demonstrated to enhance collagen gel contraction via the PI3K-PLCγ-PKC signaling pathway in immortalized human foreskin fibroblasts (BJ fibroblasts) [30] and collagen synthesis via the PI3K/Akt signaling pathway in oral mucosal fibroblasts [31]. The PDGF-mediated effects can induce fibroblast activation, transdifferentiation, migration, and accumulation at the damaged site of vessels [32,33,34]. The PDGF-mediated effects in hepatic stellate cells (HSCs) can increase cell motility through actin reorganization and cell proliferation during chronic liver injury [35]. 

### 2.3. CTGF

CTGF (also called CCN2), a protein of the CCN family, appears to be a central mediator of tissue remodeling and fibrosis induced by TGF-β [36,37]. Despite its name, CTGF is not a traditional growth factor or a cytokine because no specific receptors have been found to bind it with high affinity to induce signal transduction. Therefore, it is considered a type of matricellular protein that modulates cellular functions and the interaction of cells with the ECM by interacting with various regulatory proteins and ligands [36,37]. CTGF regulates the proliferation and differentiation of cells, as well as other biological processes, and participates in the development of inflammation, fibrosis, cancer, and other diseases [38]. Therefore, CTGF is a promising therapeutic target to treat various fibrotic diseases [39,40]. A specific monoclonal antibody (FG-3019) against CTGF recovered the impaired lung function in mice exposed to radiation, improved the reduced grip strength in rats induced by chronic overuse of the upper extremity skeletal muscle, and prevented fibrosis in both animal models [40,41]. 

### 2.4. Wnt

Wnt is a family of lipid-modified glycoprotein ligands that bind to a Frizzled family receptor that spans across the plasma membrane and interacts with the Dishevelled protein in the cytoplasm to transmit signals [42]. The canonical Wnt signaling pathway regulates the gene transcription of target genes, and the noncanonical Wnt signaling pathways regulate cell actin modification or calcium inside the cell. In the canonical Wnt signaling pathway, in the absence of Wnt, β-catenin is sequestered in the cytoplasm, phosphorylated by casein kinase 1 (CK1) and glycogen synthase kinase 3 beta (GSK3β), and degraded by the proteasome; however, in the presence of Wnt, β-catenin is stabilized and enters the nucleus, where it acts as a transcriptional coactivator that regulates the expression of various target genes. Under certain conditions, the Wnt/β-catenin signaling pathway induces EMT [43]. The Wnt/β-catenin signaling pathway also induces the gene expression of an antagonistic inhibitor, Dickkopf-1 (DKK1), and this negative feedback loop prevents the excessive activation of the Wnt signaling pathway [44]. Meanwhile, it has been reported that activation of the canonical Wnt signaling pathway is required for TGF-β-mediated fibrosis [45]. TGF-β1 suppresses DKK1 through an ERK-dependent mechanism and consequently activates the Wnt signaling pathway, which can induce cell activation and fibrosis. Therefore, the TGF-β and Wnt signaling pathways may cooperatively contribute to the development of fibrosis. 

### 2.5. HSPs

HSPs appear to play a critical role in fibrotic diseases [46]. HSPs are a group of proteins that are rapidly and highly induced by heat shock factors (HSF) in response to heat shock and other stimuli and play a critical role in the folding and refolding of proteins via their chaperone functions, especially under stressful conditions [47]. HSF-1 is sequestered in the cytoplasm by HSPs under normal conditions; however, when HSPs bind to misfolded proteins under certain stressful conditions, they release HSF-1, which then undergoes phosphorylation, trimerization, and nuclear localization to bind to the heat shock elements (HSE) found in the promoters of the HSP genes and induce the gene transcription [48]. HSP47 is a collagen-specific chaperone, and its role is essential for the structural correction of fibrillar procollagens in fibrotic diseases whose hallmark is collagen deposition [49,50,51]. HSP27 also plays a pivotal role in cell transdifferentiation to myofibroblasts, thereby providing an additional therapeutic target for the treatment of fibrotic diseases [52,53]. 

### 2.6. ROS

ROS, including superoxide anion radicals (O2^−•^), hydrogen peroxide (H_2_O_2_), hydroxyl radicals (•OH), and nitric oxide (•NO), are key modulators of TGF-β-mediated signaling pathways as well as other cell signaling pathways associated with the development and persistence of fibrosis under pathophysiological conditions [54]. In cells, a certain level of ROS is generated as a result of mitochondrial electron transport and various enzymatic reactions, such as NADPH oxidase (NOX), thereby maintaining cellular homeostasis and the redox balance; however, exposure to ultraviolet rays, radiation, toxic chemicals, drugs, or physical damage can cause ROS production beyond the cell’s antioxidant defense capacity, which leads to an emergency state in the cells [55]. As a result, pathological signs such as inflammation, cell death, and tissue remodeling appear, and the chronic consequences can cause the dysfunction of various organs. Although oxidative stress has been implicated in a wide range of diseases, the degree to which oxidative stress contributes to the pathology of the disease varies, and antioxidant therapies that target oxidative stress do not always provide satisfactory results [56]. Research projects at various stages are trying to establish treatments for different fibrotic diseases through the application of antioxidants [57,58]. 

## 3. Methods: Study Search and Selection

We used the PubMed database (https://pubmed.ncbi.nlm.nih.gov/, accessed on 9 January 2024) to search for relevant studies with the keywords: fibrosis AND rosmarinic acid. The search results provided 43 articles in total, and 42 of these articles were cited and discussed in Section 1 (2 articles), Section 4 (4 articles), and Section 5 (36 articles). We did not discuss one article that reported on the cloning of RA glycosyltransferases from *Arnebia euchroma* (Boraginaceae) [59]. 

## 4. Ex Vivo Fibrosis Model Studies

Westra et al. developed an ex vivo model of liver fibrosis induced by the prolonged culture of precision-cut liver slices (PCLSs) [60]. In rat PCLSs cultured in vitro for 48 h, the expression of PDGF-B increased, while the expression of TGF-β1 did not change. The gene expression of the fibrotic markers, such as HSP47, α-SMA, and procollagen (PCOL) 1A1, increased as well, and their gene expression was further enhanced by external PDGF-BB and TGF-β1. The early gene expression of the fibrotic markers was significantly attenuated by PDGF inhibitors (e.g., imatinib, sorafenib, and sunitinib) but was not much affected by TGF-β inhibitors (e.g., perindopril, valproic acid, RA, tetrandrine, and pirfenidone), which indicates that the PDGF signaling pathway predominates during the early stages of fibrosis development in PCLSs. 

They further developed a functional ex vivo model using precision-cut liver slices (fPCLSs) from rats with fibrotic livers after 3 weeks of bile duct ligation (BDL) [61]. In the in vitro culture of the fPCLSs for 48 h, the gene expression of the fibrotic markers (HSP47, α-SMA, and PCOL1A1) increased and the protein levels of collagen 1, mature fibrillar collagen, and total collagen increased, which indicates the continued fibrogenic process even after tissue excision. Both PDGF inhibitors (imatinib, sorafenib, and sunitinib) and TGF-β inhibitors (perindopril, valproic acid, RA, tetrandrine, and pirfenidone) significantly attenuated the gene expression of the fibrotic markers and the protein expression of collagen 1 in this model. 

In their study using PCLSs from human donors, the early onset of fibrosis during the 48 h of organ culture was demonstrated by the increases in the gene expression of HSP47 and PCOL1A1 and the collagen 1 protein level [62]. The gene expression of HSP47 and PCOL1A1 was down-regulated with sunitinib and valproic acid, and the gene expression of PCOL1A1 was also reduced by RA, tetrandrine, and pirfenidone. The TGF-β1 signaling pathway inhibitors appeared to be more effective than the PDGF signaling pathway inhibitors in suppressing fibrogenesis in the human PCLS model, which was different from the observations in the rat PCLS models. These suggest that fibrogenesis in the PCLS model may develop in different ways depending on the species and that the effects of certain treatments may also present differently. Even within the same species, the biocompatibility, anti-inflammatory, and antifibrotic effects of each treatment were different between the liver and intestine slices from humans, mice, and rats [63]. Ex vivo fibrosis model studies suggest that because the fibrogenesis process and pathology are different depending on the organ, the treatment should also be different.

## 5. In Vivo Fibrosis Model Studies: Antifibrotic Effects of RA and the Plant Extracts Containing RA

In this section, the antifibrotic effects of RA and the plant extracts containing RA are described according to their target organs. The inducer of the experimental model and treatments in each test are briefly summarized in Table 1. 

### 5.1. Liver Fibrosis

HSCs are considered one of the most important therapeutic targets for liver fibrosis because they are activated, undergo myofibroblastic transdifferentiation, and participate in the progression of the disease [95]. RA inhibited cell proliferation and the expression of TGF-β, CTGF, and α-SMA in cultured HSCs in vitro [64]. It also conferred antifibrotic effects by ameliorating the biochemical and histopathological changes in the liver and the expression of TGF-β and CTGF in mice with liver fibrosis induced by CCl_4_ [64]. 

Peroxisomal proliferator-activated receptor (PPAR) γ functions normally in quiescent HSCs, and its epigenetic repression causes cell transdifferentiation to myofibroblastic cells that mediate liver fibrosis progression [64,96]. Restored expression of PPARγ can recover the normal phenotypes of HSCs. The Wnt signaling pathway is implicated in the epigenetic repression of PPARγ, which leads to the progression of fibrosis in different organs [97]. Impeding the canonical Wnt signaling pathway using the co-receptor antagonist DKK1 abolished the epigenetic repression of PPARγ and restored the gene expression and normal phenotypes of HSCs [96].

The herbal prescription Yang-Gan-Wan, whose main active polyphenolic constituents were identified as RA and baicalin, was shown to restore the normal phenotypes of HSCs through an epigenetic mechanism [65]. Yang-Gan-Wan reduced the expression and recruitment of the DNA methyl-CpG-binding protein MeCP2 to the PPARγ promoter, the expression of histone methyltransferase, the enhancer of zeste homolog 2 (EZH2), and the methylation of histone H3 lysine 27 (H3K27), with the subsequent induction of PPARγ. RA and baicalin restored the normal phenotypes of HSCs by means of epigenetic PPARγ induction. These compounds further suppressed the expression of Wnt family members, such as Wnt10b and Wnt3a, and the canonical Wnt signaling pathway. In addition, RA treatment inhibited HSC activation and the progression of liver fibrosis in mice with cholestatic liver fibrosis induced by BDL [65]. These results suggest that RA could alleviate liver fibrosis via the suppression of the canonical Wnt signaling pathway and epigenetic de-repression of PPARγ in HSCs.

RA was shown to inhibit cell proliferation and induce apoptosis in the activated hepatic stellate cell line (HSC-T6), which correlated with the reduced phosphorylation in STAT3 and the downregulation of cyclin D1 and B cell lymphoma/leukemia (Bcl) 2 [98]. Oxidative stress plays a role in liver fibrogenesis by activating the HSCs [99]. RA inhibited the activation of matrix metalloproteinase (MMP) 2 in a nuclear factor kappa B (NF-κB)-dependent mechanism in HSC-T6 cells [100]. In addition, RA suppressed the generation of ROS and lipid peroxidation while increasing the cellular glutathione (GSH) levels and the expression of catalytic subunits of glutamate cysteine ligase (GCLc) in a nuclear factor erythroid 2-related factor 2 (NRF2)/antioxidant response element (ARE)-dependent manner. 

In Sprague Dawley (SD) rats with cholestatic liver injury induced by BDL, oral administration of the extract of *Glechoma hederacea* lowered the expression levels of TGF-β1, CTGF, SMAD2/3, and collagen and reduced the number of α-SMA-positive matrix-producing cells [66]. In addition, *Glechoma hederacea* extract attenuated the BDL-induced inflammatory cell infiltration and accumulation, the NF-κB and activator protein-1 (AP-1) activation, and the inflammatory cytokine production, whereas it inhibited the axis of the high mobility group box (HMGB) 1/Toll-like receptor (TLR) 4 intracellular signaling pathways. RA, which was identified as one of the most abundant polyphenolic compounds in *Glechoma hederacea*, also demonstrated protective effects against cholestasis-related liver injury via the attenuation of oxidative stress and the downregulation of HMGB1/TLR4, NF-κB, AP-1, and TGF-β1/SMAD signaling [67]. 

The Fuzheng Huayu recipe (FZHY) is an herbal prescription that is used in China to treat liver fibrosis [101,102]. Yang et al. identified more than eleven compounds and two metabolites in the serum from rats that were orally administered an extract of Danshen (*Salvia miltiorrhiza*), the main component of FZHY [101]. Of these compounds, salvianolic acid B (6 and 48 μM), caffeic acid (6 and 48 μM), and RA (48 μM) inhibited the proliferation of the HSC cell line (LX-2) and down-regulated the in vitro expression of α-SMA, thus supporting their contribution to the antihepatic fibrosis effects of FZHY. Plasma pharmacokinetics and tissue distribution profiles were analyzed following the oral administration of FZHY to normal and fibrotic rats induced by intraperitoneal injections of dimethylnitrosamine [102]. The results revealed that the serum levels of danshensu, salvianolic acid B, and RA were 1.69-, 2.37-, and 1.49-fold higher, respectively, in rats with liver fibrosis compared with the normal/nonfibrotic rats. It is suggested that the absorption and metabolism of active ingredients, including RA, in a mixed herbal prescription may vary depending on the disease state in the body and that they can exert therapeutic effects by modulating the relevant cells when administered at effective concentrations. 

The hepatoprotective and antifibrotic effects of RA were compared with silymarin in thioacetamide-intoxicated SD rats [68]. RA at a daily oral dose of 10 mg kg^−1^ had similar hepatoprotective effects as silymarin at a dose of 50 mg kg^−1^ in that it lowered the serum levels of alanine aminotransferase (ALT) and aspartate aminotransferase (AST), reduced the tissue levels of malondialdehyde (MDA), and increased the tissue levels of GSH in the thioacetamide-intoxicated rats. Thioacetamide increased the tissue levels of the fibrotic markers, such as TIMP-1 and hydroxyproline, in the liver, and these changes were attenuated by RA (10 mg kg^−1^) and silymarin (50 mg kg^−1^) treatment to a similar extent. Both RA and silymarin decreased the expression of collagen and α-SMA in the livers of thioacetamide-intoxicated rats. In vitro experiments using HSC-T6 cells showed that RA decreased cell viability and increased both caspase-3-positive cells and α-SMA-positive cells, thus supporting the antiproliferative and proapoptotic effects of RA in this cell type. 

When lemon balm (*Melissa officinalis*) extract and its constituent, RA, were administered orally, they were shown to alleviate nonalcoholic steatohepatitis and hepatic fibrosis in db/db mice fed a methionine- and choline-deficient (MCD) diet [69]. Oral treatments with the extract (200 mg kg^−1^) or RA (10 or 30 mg kg^−1^) alleviated liver damage that was monitored by the serum levels of ALT and AST. These treatments inhibited the accumulation of hepatic triglycerides and hydroxyproline as well as the expression of α-SMA and collagen (COL) 1A1 in the mouse model fed a MCD diet. In the associated in vitro experiments using HepG2 cells, palmitic acid caused the accumulation of lipids and triglycerides and the expression of lipogenic genes, such as sterol regulatory element-binding protein (SREBP) 1c, fatty acid synthase (FAS), and stearoyl-CoA desaturase (SCD) 1, whereas it suppressed the expression of lipolytic genes, such as carnitine palmitoyl transferase (CPT) 1L [69]. Treatment with either the extract or RA reversed these changes and restored the 5′ AMP-activated protein kinase (AMPK) phosphorylation and NRF2 activation as well as its downstream targets, including superoxide dismutase (SOD) 1 and heme oxygenase (HO) 1. 

Lyu et al. demonstrated that advanced glycation end-products (AGEs) mediate high levels of cross-linking in the ECM of cirrhotic liver tissue [70]. AGE cross-linking in the collagen matrix could be effectively inhibited by RA in vitro. Furthermore, RA inhibited the AGE-mediated cross-linking in the liver ECM and alleviated late-stage liver fibrosis, as shown by the reduced levels of collagen in the scar tissue, hepatic hydroxyproline, and the proportion of α-SMA-positive cells in male C57BL/6 mice that were treated with CCl_4_ or fed the high-fat choline-deficient L-amino acid-defined (HFCDAA) diet, thus suggesting its potential to alleviate liver cirrhosis.

### 5.2. Kidney Fibrosis

EMT is a process of cell transdifferentiation that occurs during the development of many tissues [103]. Epithelial and mesenchymal cells have different morphologies, functions, and other phenotypes; epithelial cells express high levels of E-cadherin, whereas mesenchymal cells express high levels of N-cadherin, fibronectin, vimentin, collagen, and α-SMA. EMT can occur during the early stages of wound healing, organ fibrosis, and cancer metastasis; therefore, it is important in the pathogenesis of various diseases and a useful therapeutic target [104].

WHW, a herbal prescription composed of 14 medicinal plants, has been used in oriental traditional medicine for the treatment of chronic renal failure as well as for other purposes [1,105,106]. Our previous study demonstrated that WHW extract (1 mg mL^−1^) attenuated the TGF-β1-induced morphological change of MDCKs undergoing EMT [1]. It inhibited the TGF-β1-stimulated expression of α-SMA and the phosphorylation of SMAD2. In the same study, activity-guided solvent fractionation and chromatographic separation of WHW extract identified RA as one of the main active compounds. It was further verified that RA (52–104 μM) suppressed the expression of α-SMA in MDCKs stimulated with TGF-β1 (unpublished data). In the study using cultured primary mouse macrophages and the RAW264.7 cell line, WHW extract (0.5 mg mL^−1^) suppressed the production of •NO as well as the mRNA expression and protein levels of nitric oxide synthase (NOS) 2, tumor necrosis factor (TNF) α, interleukin (IL) 1β, and IL-6 induced by lipopolysaccharide (LPS) [105]. WHW extract inhibited the phosphorylation of MAPK, such as ERK 1/2, p38, and c-Jun N-terminal kinase (JNK), and the nuclear translocation of NF-κB p65 protein in the LPS-stimulated RAW264.7 cells. These in vitro studies suggest anti-inflammatory and antifibrogenic properties of WHW extract that contains RA. 

The effect of WHW on kidney ischemia/reperfusion injury was evaluated in a mouse model [71,72]. The mice were orally administered WHW extract (0.5–100 mg kg^−1^) daily for 14 days and then subjected to 30 min of bilateral renal ischemia. Then, oral administration of WHW continued for 2 days [71]. The ischemia/reperfusion resulted in the disruption of the kidney tubular epithelial cells and increased plasma creatinine levels in the model mice, but these changes were attenuated by the WHW treatment. WHW treatment also inhibited the post-ischemic increase of H_2_O_2_ and lipid peroxidation and the post-ischemic decrease in catalase, copper/zinc SOD, and manganese SOD activity in the kidney tissue. WHW treatment enhanced the post-ischemic increase in the expression levels of HSP27 and HSP72. In another study, WHW extract was administered orally to mice for 14 days, beginning 2 days after the ischemic procedure [72]. WHW treatment attenuated the progression of renal fibrosis and prevented the post-ischemic increase of H_2_O_2_ and lipid peroxidation, as well as the decrease of copper/zinc SOD and manganese SOD activity. In addition, WHW treatment reduced the phosphorylation of ERK1/2 and JNK1/2 and the activation of NF-κB in the kidneys induced by ischemia/reperfusion. 

WHW treatment also inhibited the kidney fibrosis that was induced by unilateral ureteral obstruction (UUO) in mice [73]. Post-procedure WHW treatment (2, 10, or 50 mg kg^−1^) mitigated the UUO-induced kidney fibrosis, as determined by tubular atrophy and dilatation, collagen accumulation, interstitial space expansion, and leukocyte infiltration. WHW treatment prevented the increase in oxidative stress and decrease of catalase, copper/zinc SOD, and manganese SOD activity due to the UUO procedure. WHW reduced the expression of TGF-β1 and the phosphorylation of SMAD2/3 stimulated by the UUO procedure. Collectively, WHW extract can alleviate oxidative stress, inflammation, and fibrosis in the kidneys following an ischemia/reperfusion or UUO. 

The effects of *Elsholtzia ciliata* extract, which contains luteolin and RA as its main phenolic compounds, on renal interstitial fibrosis were examined in male SD rats with UUO [74]. Orally administered extract (300 or 500 mg kg^−1^) reduced UUO-induced renal damage, histopathological alterations, and interstitial fibrosis. In addition, the renal expression levels of TNF-α, TGF-β1, and SMAD3 were reduced in the extract-treated rats compared to the untreated UUO model rats. The accompanying in vitro studies revealed that this extract (200 μg mL^−l^) ameliorated the LPS-induced overexpression of NF-κB, TNF-α, and IL-6, improved oxidative stress in RAW 264.7 cells, and suppressed the TGF-β-induced expression of α-SMA and MMP9 in human renal mesangial cells. Thus, *Elsholtzia ciliata* extract may impede the inflammatory and fibrogenic signaling pathways that cause renal fibrotic disease.

The inhibitory effects of RA on kidney fibrosis and EMT were examined using a UUO mouse model and an indoxyl sulfate-stimulated NRK-52E cell model [75]. C57BL/6 mice were orally administered RA (10 or 20 mg kg^−1^) before and after UUO surgery. UUO caused kidney damage, which was determined by the increase in serum creatine and blood urea nitrogen (BUN) levels, and the damage was reduced by RA treatment. The UUO-induced increase of α-SMA, collagen I, fibronectin, and vimentin and the decrease of E-cadherin were attenuated by RA treatment. NRK-52E cells were treated with 4 mM indoxyl sulfate with or without RA (20 or 40 μM). The indoxyl sulfate-induced proliferation and migration of the cells were inhibited by RA treatment. RA treatment further attenuated the increase of α-SMA, collagen I, fibronectin, vimentin, and TGF-β1 and the decrease of E-cadherin induced by indoxyl sulfate. RA treatment inhibited the phosphorylation of Akt in both the cell and animal models. Thus, RA was suggested to ameliorate renal interstitial fibrosis by inhibiting the Akt-mediated EMT.

RA was shown to attenuate the nephrotoxicity of cadmium both in vitro and in vivo [76]. RA prevented the apoptotic death of cultured mouse proximal tubular epithelial cells that were exposed to CdCl_2_. It attenuated the CdCl_2_-induced increased production of ROS/•NO, lipid peroxidation, protein carbonylation, the increased activity of NOX activity, and the decreased levels of coenzyme 9, coenzyme 10, and GSH, as well as the decreased activity of SOD, catalase, glucose-6-phosphate dehydrogenase (G6PDH), glutathione peroxidase (GPX), glutathione S-transferase (GST), and glutathione reductase (GR). CdCl_2_ stimulated apoptotic events that were mediated by Bcl-2, Bcl-2-associated death protein (Bad), apoptotic protease activating factor (Apaf) 1, cytochrome C, and caspases 3, 8, and 9, and the fibrogenic events that were accompanied by the upregulation of α-SMA, E-cadherin, and collagen IV in the cells, and all these events were attenuated by RA treatment. Swiss albino mice were treated with CdCl_2_ (4 mg kg^−1^) with or without RA treatment (50 mg kg^−1^), and the pathological changes were monitored by assessing the blood, urine, and tissues. The biochemical and histological data supported the pharmacological effects of RA attenuating oxidative stress, apoptosis, inflammation, and fibrosis in the kidneys caused by cadmium exposure. 

The effects of *Lycopus lucidus* extract, which contains caffeic acid, luteolin-7-O-β-D-glucoside, and RA as the main phenolic compounds, on kidney fibrosis were examined in rhTGF-β1-stimulated SV40 MES13 cells and a streptozotocin-induced diabetic nephropathy rat model [77]. Treatment with the extract (50–400 μg mL^−1^) suppressed the activation of SMAD2 and ERK1/2 and downregulated TGF-βRI, TGF-βRII, SMAD4, and SMAD7 in TGF-β1-stimulated cells. This extract (3, 6, or 12 g kg^−1^) inhibited SMAD2 phosphorylation, reduced TGF-β1 expression, ameliorated the expansion of the mesangial area in glomerular tissue, reduced the levels of serum creatine and BUN, and reduced the total SOD activity in the streptozotocin-induced diabetic nephropathy rats. Thus, *Lycopus lucidus* extract is considered to be able to inhibit renal fibrosis by impeding the TGF-β signaling pathway and attenuating diabetic nephropathy. 

Guanxinning injection, a herbal prescription composed of Radix et Rhizoma Salviae Miltiorrhizae (*Salvia miltiorrhiza*) and Chuanxiong Rhizoma (*Ligusticum chuanxiong*), was tested for its effects on renal fibrosis in mice with heart failure induced by transverse aortic constriction [78]. Guanxinning injection was administered via tail vein at doses of 3–12 mL kg^−1^, and it relieved the cardiac function indexes (ejection fraction, cardiac output, and left ventricle volume), kidney functional indexes (serum creatinine), and kidney fibrosis indexes (collagen volume fraction and CTGF) in the model mice. Guanxinning injection further increased catalase, GPX4, the x(c)(-)cysteine/glutamate antiporter SLC7A11, and ferritin heavy chain 1, while decreasing xanthine oxidase and NOS in the kidneys. Its major active ingredients include RA, caffeic acid, ferulic acid, etc. Thus, Guanxinning injection could help maintain cardiac function and alleviate the progression of fibrosis in the kidneys due to heart failure through the regulation of redox metabolism.

Patients with autosomal dominant polycystic kidney disease had higher expression levels of complement factor B and complement component 9 in the kidneys compared with control subjects or patients with other types of chronic kidney disease [107]. Oral administration of RA (300 mg kg^−1^, daily) for 4 weeks reduced serum creatine, BUN, kidney weight, cyst index, fibrosis, expression of complement factor B and C5b-9, and the number of Ki67-positive nuclei, as well as the inflammatory cells, without affecting the body weight in Pkd1^−/−^ knockout mice. This supports the therapeutic potential of RA as an inhibitor of the complement pathway associated with autosomal dominant polycystic kidney disease progression [107].

### 5.3. Heart Fibrosis

The ethanolic extract of the aerial parts of basil (*Ocimum basilicum*), which contains RA as the principal phenolic compound, has been shown to improve cardiac function and inhibit the histopathological changes in response to isoproterenol-induced myocardial infarction (MI) in male Wistar albino rats [79]. Treatment with isoproterenol (100 mg kg^−1^) daily for 2 consecutive days caused an elevation in the ST segment in the electrocardiogram, indicative of a MI, a reduction in the left ventricular contractility, an increase in the left ventricular end-diastolic pressure, and an increase in myocardial necrosis and fibrosis, all of which were significantly attenuated by the subcutaneous injection of 10, 20, or 40 mg kg^−1^ of the extract twice per day. In addition, the isoproterenol-induced elevated MDA levels in the serum and myocardium were suppressed by the extract, thus suggesting that the cardioprotective effects of *O. basilicum* extract could be associated with its antioxidant activity.

The aqueous extract of Xia-Ku-Cao (*Prunella vulgaris*), as well as its caffeic acid, ursolic acid, and RA components, exhibited a cardioprotective effect in acute MI in male SD rats with left anterior descending coronary artery (LAD) ligation [80]. The extract (400 mg kg^−1^) administered by intragastric gavage after surgery improved cardiac function and reduced the infarct size, inflammation, fibrosis, oxidative damage, and apoptosis of cardiomyocytes. Phenolic compounds, such as caffeic acid (400 mg kg^−1^), ursolic acid (400 mg kg^−1^), and RA (400 mg kg^−1^), improved cardiac function and suppressed inflammatory aggregation and fibrosis in MI rat models. RNA-seq analysis and an additional in vitro study suggest that the therapeutic effect of *P. vulgaris* extract and its phenolic compounds might be related to the reduced expression of NOD-like receptor protein 3 (NLRP3), which is presumed to play a critical role in the inflammatory process during cardiac remodeling after a MI [108].

RA demonstrated antifibrotic effects in male SD rats with a MI induced by LAD ligation surgery [81]. The oral administration of RA (50, 100, or 200 mg kg^−1^) ameliorated the infarct size and MI-induced changes in the left ventricular systolic pressure, +dp/dt_max_, and −dp/dt_max_. RA attenuated the cardiac fibrosis, as determined by the collagen volume fraction and the expression of collagen I, collagen III, α-SMA, and hydroxyproline. RA treatment also decreased the expression of angiotensin-converting enzyme (ACE), angiotensin type 1 receptor (AT1R), and phospho-p38 MAPK while increasing the expression of ACE2, which may be associated with the cardioprotective effects of RA against cardiac dysfunction and fibrosis. 

An injectable hydrogel encapsulating polydopamine-RA nanoparticles with dual responsiveness to pH and ROS was developed to achieve on-demand drug release in the MI microenvironment [82]. Oxidized xanthan gum grafted with 3-aminophenylboronic acid and dopamine-grafted gelatin were combined to prepare an “OGD” hydrogel. The polypyrrole-modified gelatin was combined with “OGD” to prepare an “OGDP” hydrogel. The polydopamine-RA nanoparticles were added to the “OGD” and “OGDP” hydrogels to prepare the “OGDR” and “OGDPR” hydrogels. Rats were subjected to LAD ligation to induce MI, and on day 2 after modeling, rats with a confirmed MI were subjected to multiple injections with the “OGD”, “OGDR”, “OGDP”, or “OGDPR” hydrogels in and around the infarct area. The OGDPR hydrogel showed the best results by exerting anti-inflammatory, antiapoptotic, and antifibrotic effects. The multifunctional hydrogel promoted the expression of heart-specific markers, thus restoring heart function after a MI.

RA also attenuated cardiac remodeling and fibrosis following a long-term pressure overload in male C57/B6 mice subjected to aortic banding to generate pressure overload, which led to cardiac dysfunction with reduced fractional shortening [83]. Orally administered RA (100 mg kg^−1^) attenuated the fibrotic response and cardiac dysfunction after pressure overload without affecting the hypertrophic response. Aortic banding increased the expression of collagen I and III, CTGF, fibronectin, TGF-β1, and α-SMA, and the phosphorylation of ERK, Akt, p38, AMPK, and acetyl-CoA carboxylase (ACC). RA attenuated the increased expression of collagen I and III, CTGF, fibronectin, TGF-β1, and α-SMA and enhanced the increased phosphorylation of AMPK and ACC without significantly affecting the phosphorylation of ERK, Akt, and p38. The cardioprotective effects of RA in reducing the expression of collagen I and III, the phosphorylation of SMAD, and the restored fractional shortening induced by aortic banding were abolished in AMPKα2 knockout mice. 

RA has been demonstrated to alleviate the apoptosis of cardiomyocytes [109] and cardiac fibrosis induced by doxorubicin, an anticancer medicine, in male rats [84]. Doxorubicin (2 mg kg^−1^ per 48 h) increased the heart-to-body weight ratio, reduced the heart rate and blood pressure, and caused fibrosis and necrosis of the cardiac tissue [84]. These toxic effects of doxorubicin were attenuated by the administration of RA (10, 20, or 40 mg kg^−1^). RA administration also attenuated the doxorubicin-induced increase of MDA and decrease of GSH in the heart tissue, thereby implying that its antioxidant properties underlie the protective effects against doxorubicin-induced cardiotoxicity. 

### 5.4. Lung Fibrosis

Rosemary (*Rosmarinus officinalis*) leaf extracts, which contain carnosic acid and RA, have been shown to inhibit bleomycin-induced pulmonary fibrosis [85]. Male Wistar rats were given a single dose of bleomycin (4 mg kg^−1^, intratracheal), and the extract (75 mg kg^−1^) was administered 3 days later and continued for 4 weeks (curative group), or administered 2 weeks before bleomycin administration and continued for 15 days thereafter (prophylactic group). The lung architecture was more retained in the curative and prophylactic groups compared to the bleomycin group, which was associated with lower fibrosis scores (2.33 and 1.8 vs. 3.7 of the bleomycin group, respectively), reduced MDA levels (141% and 108% vs. 258% of the normal value, respectively), high catalase levels (103% and 117% vs. 59% of the normal value, respectively), and enhanced GST activity (85% and 69% vs. 23% of the normal value, respectively). The antifibrotic and antioxidant effects of carnosic acid, RA, and their combination were verified in the bleomycin-induced fibrosis model in rats [86]. In addition, purified rosemary leaf extract with increased RA and carnosic acid contents and decreased essential oil content exhibited potent antifibrotic efficacy in an animal model [110].

RA attenuated the pulmonary fibrosis caused by irradiation. Treatments of male SD rats with RA (30, 60, or 120 mg kg^−1^) before 15 Gy of X-ray irradiation decreased the expression of inflammatory mediators, NF-κB phosphorylation, and the production of ROS [87]. RA inhibited Ras homolog family member A (RhoA)/Rho-associated protein kinase (Rock) signaling through the upregulation of the microRNA (miR) 19b-3p, which targets the myosin phosphatase target subunit 1 (MYPT1) and leads to the inhibition of TGF-β1 signaling and subsequent fibrosis [87]. 

*Salvia miltiorrhiza* extract, which contains salvianic acid A and B, caffeic acid, RA, and tanshinone IIA, attenuated ovalbumin-induced asthma in mice [88]. The ovalbumin challenge increased airway resistance, inflammatory cell infiltration, Th1/Th2 cytokine levels in the bronchoalveolar lavage fluid (BALF), cell hyperplasia, collagen deposition, and airway wall thickening. Daily treatment with the water extract of *S. miltiorrhiza* (156 mg kg^−1^) significantly alleviated these pathological changes. In the associated in vitro experiments, caffeic acid and RA potently reduced E-cadherin and vimentin in the TGF-β1-induced BEAS-2B cells and α-SMA and COL1A1 in the TGF-β1-induced MRC-5 cells. RA also attenuated the immunological and inflammatory responses and normalized the pathological features in the lungs of ovalbumin-induced asthmatic rats [89]. The asthmatic rats showed increased levels of IL-4, immunoglobulin (Ig) E, phospholipase A (PLA) 2, and total protein in the BALF and decreased levels of interferon (IFN) γ and the IFN-γ/IL-4 ratio compared to the control group. The RA treatment reduced the levels of IL-4, IgE, PLA2, and total protein and restored the IFN-γ/IL-4 ratio. 

### 5.5. Post-Surgical Abdominal Adhesion

Peritoneal adhesion occurs when the peritoneum is damaged as a result of various injury events, including abdominal surgery [111]. Peritoneal bands connect the peritoneum to different visceral organs and cause abdominal discomfort, infertility in women, intestinal blockage, and other ailments [112]. RA (50 or 70 mg kg^−1^) inhibited the formation of postoperative peritoneal adhesion in male Wistar albino rats [90]. RA decreased the peritoneal adhesion score and was associated with reduced fibroblast proliferation, the expression of TGF-β1 (a fibrotic marker), TNF-α (an inflammation marker), and vascular endothelial growth factor (VEGF) (an angiogenesis marker), and reduced MDA levels (an oxidative stress marker). A polygalacturonic acid-conjugated cysteine hydrogel has been developed as a drug carrier for RA, and animal implant studies have shown that hydrogel films with and without RA reduce the incidence of post-surgical adhesion and early inflammatory reactions [91].

### 5.6. Fibrosis in the Salivary Glands

The three major types of salivary glands are the parotid, submaxillary, and sublingual glands [113]. The salivary glands have a high risk of exposure to oxidative damage after irradiation therapy for head and neck cancers [114]. The radioprotective effect of RA on the parotid gland was examined in SD rats irradiated with 15 Gy X-ray irradiation [92]. RA treatment (60 and 120 mg kg^−1^) attenuated the radiation-induced hyposalivation and oxidative stress in the parotid gland as effectively as amifostine (250 mg kg^−1^). The radiation increased NOX 4 and decreased PPARγ coactivator (PGC) 1α, which were normalized by RA treatment. RA reduced apoptosis by inhibiting p53/JNK activation and fibrosis by downregulating inflammatory factor levels. Therefore, RA has the therapeutic potential to treat radiation-induced parotid gland injuries.

### 5.7. Skin Wounds

The skin wound healing effects of RA were examined in Wistar albino rats with a 2 cm full-thickness skin wound [93]. The topical application of a 10% RA cream enhanced the wound size reduction more effectively compared to a 5% dexpanthenol cream application or nontreatment, which resulted in fewer scars. An oxidized dextran/amidated gelatin hydrogel, “ODex-AG”, and an RA-grafted hydrogel, “ODex-AG-RA”, were developed as dressings for skin injuries [94]. Both the “ODex-AG” and “ODex-AG-RA” hydrogels promoted skin wound healing in a rat model of a full-thickness skin wound. The “ODex-AG-RA” hydrogel effectively enhanced the collagen deposition and neovascularization (CD31) in the wounds and alleviated inflammation (TNF-α and CD163) and oxidative stress (MDA and H_2_O_2_) compared to the “ODex-AG” hydrogel. Overall, this study demonstrated the utility of RA-grafted hydrogels as a wound dressing with anti-inflammatory and antioxidant activities.

### 5.8. Pterygium in the Eyes

Pterygium occurs due to the uncontrolled excessive proliferation of epithelial tissue in the eyes, and the lesion can invade the cornea, potentially causing irregular corneal astigmatism and vision loss [115]. The antifibrotic effect of RA has been tested in pterygium epithelial cells [116]. RA treatment (100 µM) significantly decreased the cell viability and the protein expression of type I collagen, TGF-β1, TGF-βRII, phospho-SMAD1/5, phospho-SMAD2, phospho-SMAD, and SMAD4. Thus, RA is suggested to inhibit the TGF-β1/SMAD signaling pathway involved in pterygium epithelial cell-mediated fibrosis. Further studies are expected to evaluate the therapeutic potential of RA for the in vivo treatment of pterygium.

### 5.9. Fibrosis of Autologous Fat Grafts

After the autologous transplantation of inguinal fat pads to the parascapular area in SD rats, an ethanol solution of RA was intraperitoneally injected (20 mg kg^−1^) daily for a week and then weekly for 7 weeks [117]. The total oxidant status, MDA, and the expression levels of TNF-α and TGF-β1 were lower in the transplanted tissue of the RA-treated group compared to the control or vehicle (ethanol)-treated groups, which indicates that RA could reduce the necrosis, inflammation, and fibrosis of fat grafts [117].

## 6. Lamiaceae Plants with Antifibrotic Effects

Herbal remedies that have been used to treat fibrotic diseases in traditional medicine are mostly mixtures of multiple plants. Therefore, the question arises as to which compound of which plant is responsible for its efficacy. In previous research, the activity-guided solvent fractionation and chromatographic separation of WHW led to the identification of RA as the active compound [1]. Interestingly, all plants whose single extract was reported to contain RA and to show antifibrotic activity belong to the family Lamiaceae (Table 2). These include *Glechoma hederacea*, *Melissa officinalis*, *Elsholtzia ciliata*, *Lycopus lucidus*, *Ocimum basilicum*, *Prunella vulgaris*, *Salvia rosmarinus* (*Rosmarinus officinalis*), and *Salvia miltiorrhiza*. 

Traditional herbal prescriptions, such as FZHY, WHW, and Guanxinning injections, commonly include Lamiaceae plants, i.e., *Salvia miltiorrhiza* and *Perilla frutescens* (Table 2). This suggests that RA is the active ingredient for the antifibrotic effect of Lamiaceae plants, and these plants are a valuable source of RA. *Perilla frutescens* also contains RA [119], and the therapeutic effects of the extracts of this plant and its polyphenolic components on chronic kidney disease have been investigated [120]. Meanwhile, Yang-Gan-Wan contains RA as one of its main compounds, even though this herbal prescription does not include Lamiaceae plants, and thus RA is presumed to have originated from Apiaceae plants. 

Lamiaceae plants commonly contain RA, which means that they share a biosynthetic pathway for the generation of this compound [121]. The RA biosynthesis starts with the condensation of 4-coumaroyl-CoA and (R)-3-(4-hydroxyphenyl)lactate to produce 4-coumaroyl-(R)-3-(4-hydroxyphenyl)lactate. This compound is further oxidized via either 4-coumaroyl-(R)-3-(3,4-dihydroxyphenyl)lactate or caffeoyl-(R)-3-(4-hydroxyphenyl)lactate into RA, caffeoyl-(R)-3-(3,4-dihydroxyphenyl)lactate. Structurally similar compounds, such as metabolites of the RA synthesis pathway, may be contained in the plants of the Lamiaceae family and exhibit biological activity similar to RA in animal systems. Therefore, RA is not necessarily the only active ingredient. Furthermore, it should be noted that RA synthesis is not limited to Lamiaceae plants [121].

## 7. Key Mediators of the Antifibrotic Effects of RA

**Figure 3:**
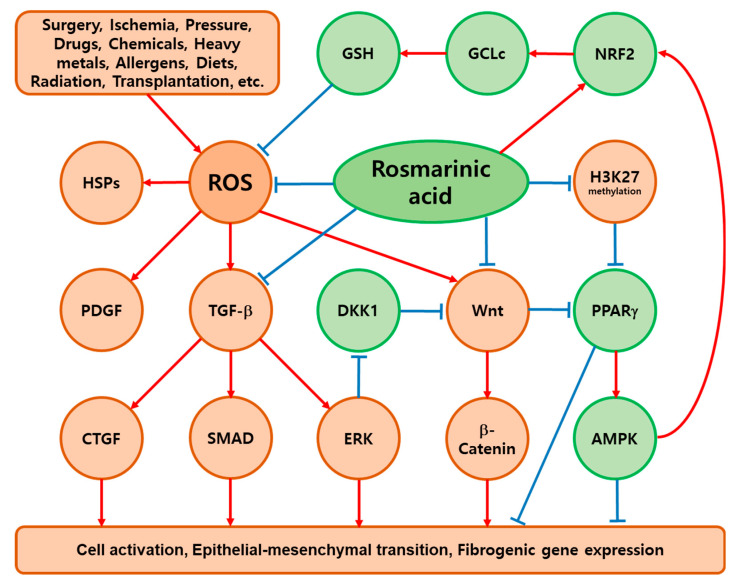
Hypothetical mechanisms of the antifibrotic action of rosmarinic acid (RA). RA can remove ROS and suppress cell activation, EMT, and fibrogenic gene expression by inhibiting the stress-induced expression of TGF-β, CTGF, and Wnt and their signaling pathways. Peroxisomal proliferator-activated receptor (PPAR) γ is repressed in myofibroblastic cells, and RA restores the gene expression of PPAR γ through an epigenetic mechanism that reduces the methylation of histone H3 lysine 27 (H3K27). PPARγ activates 5′ AMP-activated protein kinase (AMPK), which inhibits cell activation, EMT, and fibrogenic gene expression. RA also activates the nuclear factor erythroid 2-related factor (NRF) 2 via multiple mechanisms to induce the transcriptional expression of phase II/antioxidant enzymes, and the increased expression of catalytic subunits of glutamate cysteine ligase (GCLc) promotes the synthesis of glutathione (GSH), thereby reducing ROS. Collectively, RA alleviates oxidative stress and maintains the structural and functional integrity of the organs through multiple mechanisms. Profibrotic and antifibrotic factors are shown in brown and green circles, respectively. Sharp red arrows (→) indicate stimulation, and blunt blue arrows (⊥) indicate suppression.

Many ex vivo and in vivo studies have supported that RA and several plant extracts have antifibrotic effects, attenuating cell activation, EMT, and the expression of fibrogenic genes. RA is considered to regulate multiple targets beyond the antioxidant effect of nonselectively relieving oxidative stress. RA could modulate common mediators of fibrosis, such as TGF-β, PDGF, CTGF, Wnt, HSPs, etc. In addition, PPARγ, AMPK, NF-κB, and NRF2 appear to be additional key mediators of the antifibrotic effects of RA, as discussed below. Based on the considerations and discussions in this review, the mechanism of antifibrotic action of RA is schematized in Figure 3. 

### 7.1. PPARγ

The nuclear receptor superfamily PPARα, β/δ, and γ is known to regulate cellular metabolic energy homeostasis [122,123]. The ligand-activated transcription factor PPARγ forms a heterodimer with the retinoid X receptor (RXR) and binds to the PPAR response elements (PPREs), which are mainly identified in the promoters of genes involved in the metabolism of lipids and glucose [124]. PGC-1α binds PPARs and RXRs to co-activate the expression of target genes [125]. The activation of PPARγ appears to inhibit the canonical and noncanonical TGF-β-mediated signaling pathways, thereby supporting the cross-talk between the PPAR and TGF-β superfamilies [126]. The herbal prescription Yang-Gan-Wan and its constituents, RA and baicalin, induced the gene expression of PPARγ in HSCs [65]. RA treatment restored the gene expression of PPARα and PGC-1α that were suppressed by palmitic acid in HepG2 cells [69]. RA enhanced AMPKα phosphorylation and suppressed α-SMA expression in cardiac fibroblasts induced by TGF-β1, and the RA effects were abolished by the small interfering RNA (siRNA)-mediated depletion of PPARγ or its pharmacological inhibition with GW9662, a specific PPARγ antagonist [83]. Thus, it is suggested that PPARα, PPARγ, and PGC-1α are important mediators of the antifibrotic effects of RA.

### 7.2. AMPK 

AMPK is a heterotrimeric protein complex that consists of α, β, and γ subunits and plays a role in maintaining cellular energy homeostasis [127]. It is allosterically activated by the binding of AMP to the γ subunit and deactivated upon the displacement of AMP by ATP [128]. It is also activated through the phosphorylation of the α subunit by liver kinase (LK) B1, calcium/calmodulin-dependent protein kinase (CAMK) 2, or TGFβ-activated kinase (TAK) 1 and deactivated through the dephosphorylation by several protein phosphatases [129]. The AMPK-mediated phosphorylation of ACC1 or SREBP1c suppressed the synthesis of fatty acids, cholesterol, and triglycerides while increasing the uptake and β-oxidation of fatty acids [129]. RA and RA-enriched lemon balm extract increased the phosphorylation of AMPK, its upstream protein kinases, such as LKB1 and CAMK2, and its downstream substrate, ACC1, in HepG2 cells [69]. RA also enhanced the phosphorylation of AMPK and ACC that were induced by pressure overload in mice, and the antifibrotic and cardioprotective effects of RA were abolished in AMPKα2 knockout mice [83]. RA treatment increased the phosphorylation of AMPKα and ACC in cardiac fibroblasts and suppressed the TGF-β1-induced α-SMA expression, which was abolished by the short hairpin RNA (shRNA)-mediated knockdown of AMPKα2 [83]. Thus, AMPK activation is a key mechanism for the antifibrotic effects of RA.

### 7.3. NRF2 

NRF2 is a transcription factor that binds to AREs, which are commonly found in the promoter region of phase II metabolism/antioxidant enzymes [130,131]. In the base state, Kelch-like ECH-associated protein (KEAP) 1 and β-transducin repeat-containing protein (TrCP) sequester NRF2 in the cytosol and promote its proteasomal degradation. However, when their interaction is weakened by ROS, electrophilic substances, or protein kinases, NRF2 is released from KEAP1 or β-TrCP and enters the nucleus to induce the gene expression of target genes, such as GCLc, catalase, SOD, and HO-1 [132,133]. 

RA suppressed the generation of ROS while increasing the cellular GSH levels in HSC-T6 cells [100]. RA stimulated the nuclear translocation of NRF2 and the activation of AREs in the promoters, which led to the expression of the target genes, such as GCLc, which is involved in GSH synthesis. The shRNA-mediated knockdown of NRF2, but not NF-κB, abolished the RA-induced decrease of ROS levels and increase of GSH levels in the cells. RA restored the protein levels of NRF2 and its downstream targets, including SOD1 and HO-1, in palmitic acid-treated HepG2 cells [69]. RA increased GSH levels in the cultured mouse proximal tubular epithelial cells exposed to CdCl_2_ [76]. This effect of RA has also been observed in the livers of thioacetamide-intoxicated rats [68] and the hearts of doxorubicin-exposed rats [84]. Thus, RA can enhance cellular antioxidant capacity and resistance to oxidative stress via the NRF2/ARE-mediated gene expression of antioxidant enzymes.

### 7.4. NF-κB

NF-κB is a family of inducible transcription factors that mediate the transcription of target genes by binding to a specific DNA element and κB enhancer in a variety of immune and inflammatory processes [134]. The canonical NF-κB pathway responds to diverse signals transmitted through members of the TNF receptor (TNFR) superfamily as well as other relevant receptors. The NF-κB proteins, such as NF-κB1 (p50), are normally sequestered in the cytoplasm by inhibitory proteins, such as IκBα. However, under stimulated conditions, IκBα undergoes site-specific phosphorylation by an IκB kinase (IKK) complex and ubiquitin-dependent proteasomal degradation, resulting in the rapid and transient nuclear translocation of the NF-κB1 (p50)/RelA (p65) dimer, which transactivates the expression of the target gene [134]. The noncanonical NF-κB pathways are regulated by different mechanisms [135]. Accumulating evidence suggests that the dysregulation of NF-κB activity may be associated with various inflammatory diseases and cancers, and thus NF-κB has been one of the most studied targets for the therapy of diseases [136]. 

RA has been shown to induce the nuclear translocation of NF-κB and inhibit the expression of MMP2 in HSC-T6 cells [100]. The inhibitory effects of RA on the expression of MMP-2 were abolished by the shRNA-mediated depletion of NF-κB or NRF2. Thus, it is suggested that RA may impede the progression of HSC activation through NF-κB-dependent mechanisms. On the other hand, RA reduced the activation of the NF-κB pathway in mouse tubular epithelial cells exposed to CdCl_2_ [76]. RA and *Glechoma hederacea* extract reduced NF-κB activity in rat livers with cholestasis [66,67]. WHW attenuated the activation of NF-κB in RAW264.7 cells that were stimulated with LPS [105] and in the kidneys of mice exposed to ischemic surgery [72]. *Elsholtzia ciliata* extract also attenuated NF-κB activation in LPS-stimulated RAW264.7 cells [74]. Therefore, NF-κB is an important mediator in both the pathogenesis and treatment of diseases, and it is proposed that RA and plant extracts containing RA have a therapeutic effect by maintaining the NF-κB activity at an appropriate level. 

## 8. Future Perspectives 

RA and the extracts of the Lamiaceae plants that contain RA have various biological activities and a high potential to be used as nutraceuticals to improve human health or as medicines for the treatment of diseases. As explored in this review, they affect various signaling pathways and biological events in cells and animal models that are associated with fibrotic diseases of different organs. In addition, bioengineering technologies are being developed to increase the RA content in plants and RA production efficiency [137,138]. Furthermore, research on formulations, such as nanoparticles and hydrogels, that increase RA bioavailability or its pharmacological efficacy is also emerging [82,91,94,139,140]. Advances in these related fields will accelerate the industrial and medical utilization of RA. Information regarding the safety and pharmacokinetics of RA has been previously reported [141,142]. We expect that the development of advanced medicines specialized for fibrotic diseases in human organs based on the pharmacological properties of RA is a step further from the traditional use of plant extracts and herbal prescriptions. 

We present several challenges for future studies, as follows:

- To verify key mediators of RA effects as therapeutic targets for fibrotic diseases.
- To compare RA with other competing substances in terms of efficacy and safety.
- To discover new drug candidates using RA as a lead compound.
- To develop formulations to enhance the bioavailability and targeted delivery of RA.
- To evaluate the safety and therapeutic efficacy of RA through clinical trials.
- To expand the industrial and medical applications of RA.

## 9. Conclusions

**Figure 4:**
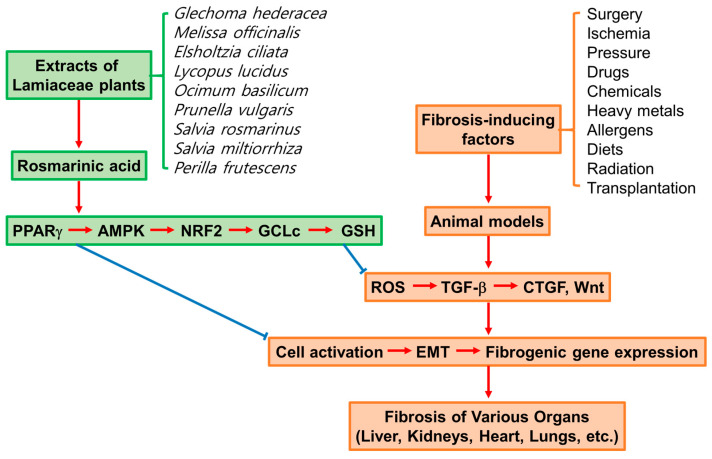
Inhibitory effects of rosmarinic acid (RA) and Lamiaceae plant extracts on fibrosis of various organs in animal models exposed to various fibrosis-inducing factors. Profibrotic and antifibrotic factors are shown in brown and green boxes, respectively. Sharp red arrows (→) indicate stimulation or progression, and blunt blue arrows (⊥) indicate inhibition or suppression.

In conclusion, accumulating scientific evidence supports the effectiveness of RA and Lamiaceae plant extracts in alleviating fibrosis in various organs, including the liver, kidneys, heart, lungs, etc., as summarized in Figure 4. 

RA modulates multiple signaling pathways mediated by PPARγ, AMPK, NRF2, and/or NF-κB in cells. It enhances cellular antioxidant capacity, alleviates ROS-mediated oxidative stress and inflammation, inhibits the TGF-β, CTGF, and Wnt-mediated signaling pathways, and suppresses cell activation, EMT, and fibrogenic gene expression in various organs, contributing to the recovery of structural and functional integrities in each organ. 

Although RA-containing plant extracts are already used in traditional medicine, it is hoped that RA will be developed into more advanced nutraceuticals or pharmaceuticals based on updated information on its efficacy, mechanism of action, safety, and pharmacokinetics. For these industrial and medical applications, well-designed clinical trials on the efficacy and safety of RA-based preparations are needed.

## Figures and Tables

**Table 1:** Animal studies on the protective effects of rosmarinic acid (RA) and plant extracts containing RA against fibrosis in different disease models.

Disease Model	Animals	Inducer	Test Materials and Doses	Literature
Liver fibrosis	Sprague Dawley rats	CCl_4_	RA, 2.5, 5, or 10 mg kg^−1^, i.g.	[64]
Cholestatic liver fibrosis	C57BL/6 mice	Bile duct ligation	RA, 4 mg kg^−1^, i.p	[65]
Cholestatic liver injury	Sprague Dawley rats	Bile duct ligation	*Glechoma hederacea* extract, 500 or 2000 mg kg^−1^, p.o.	[66]
			RA, 5 or 20 mg kg^−1^, p.o.	[67]
Liver fibrosis	Sprague Dawley rats	Thioacetamide	RA, 10 mg kg^−1^, p.o.; silymarin, 50 mg kg^−1^, p.o.	[68]
Nonalcoholic steatohepatitis	db/db mice	Methionine- and choline-deficient diet	Lemon balm (*Melissa officinalis*) extract, 200 mg kg^−1^, p.o.; RA, 10 or 30 mg kg^−1^, p.o.	[69]
Liver cirrhosis	C57BL/6 mice	CCl_4_ or high-fat choline-deficient L-amino acid-defined diet	RA, 30 mg kg^−1^, i.g.	[70]
Kidney ischemia/reperfusion injury	BALB/c mice	Bilateral renal ischemia	Wen-pi-tang-Hab-Wu-ling-san, 0.5, 2, 10, 50, or 100 mg/kg^−1^, p.o., pre- and post-ischemic treatments.	[71]
			Wen-pi-tang-Hab-Wu-ling-san, 10 or 100 mg kg^−1^, p.o.	[72]
Kidney fibrosis	C57BL/6 mice	Unilateral ureteral obstruction	Wen-pi-tang-Hab-Wu-ling-san, 2, 10, or 50 mg kg^−1^, p.o.	[73]
Kidney fibrosis	Sprague Dawley rats	Unilateral ureteral obstruction	*Elsholtzia ciliata* extract, 300 or 500 mg kg^−1^, p.o.; captopril, 200 mg kg^−1^, p.o.	[74]
Kidney fibrosis	C57BL/6 mice	Unilateral ureteral obstruction	RA, 10 or 20 mg kg^−1^, o.g.	[75]
Nephrotoxicity	Swiss mice	CdCl_2_	RA, 50 mg kg^−1^, p.o.	[76]
Diabetic nephropathy	Sprague Dawley rats	Streptozotocin	*Lycopus lucidus* extract, 3, 6, or 12 g kg^−1^	[77]
Kidney fibrosis in heart failure	C57BL/6 mice	Transverse aortic constriction	Guanxinning injection, 3, 6, or 12 mL kg^−1^, i.v.; telmisartan, 6.1 mg/kg, o.g.	[78]
Myocardial infarction	Wistar rats	Isoproterenol	Basil (*Ocimum basilicum*) extract, 10, 20, or 40 mg kg^−1^, p.o.	[79]
Myocardial infarction	Sprague Dawley rats	Left anterior descending coronary artery ligation	Xia-Ku-Cao (*Prunella vulgaris*) extract, 200 or 400 mg kg^−1^, p.o.	[80]
Myocardial infarction	Sprague Dawley rats	Left anterior descending coronary artery ligation	RA, 50, 100, or 200 mg kg^−1^, p.o.	[81]
Myocardial infarction	Sprague Dawley rats	Left anterior descending coronary artery ligation	Hydrogel encapsulating RA nanoparticles, 100 μL per animal heart, s.c.	[82]
Long-term pressure overload	C57BL/6 mice	Aortic banding	RA, 100 mg kg^−1^ day^−1^, i.g.	[83]
Cardiotoxicity	Wistar rats	Doxorubicin	RA, 10, 20, or 40 mg kg^−1^, i.p.; vitamin E, 200 mg kg^−1^, i.p.	[84]
Lung fibrosis	Wistar rats	Bleomycin	Rosemary (*Rosmarinus officinalis*) leaf extract, 75 mg kg^−1^, i.p.	[85]
Lung fibrosis	Wistar rats	Bleomycin	RA, 5 mg kg^−1^, i.p.; carnosic acid, 5 mg kg^−1^, i.p.; vitamin E, 300 mg kg^−1^, i.p.	[86]
Lung fibrosis	Sprague Dawley rats	X-ray irradiation	RA, 30, 60, or 120 mg kg^−1^, i.g.	[87]
Asthma	BALB/c mice	Ovalbumin	*Salvia miltiorrhiza* water extract, 31.5 or 156 mg kg^−1^, p.o.; *Salvia miltiorrhiza* ethanol extract, 49.2 or 246 mg kg^−1^, p.o.	[88]
Asthma	Wistar rats	Ovalbumin	RA, 0.125, 0.250, or 0.500 mg mL^−1^ in drinking water; dexamethasone, 1.25 g mL^−1^ in drinking water.	[89]
Peritoneal adhesion	Wistar rats	Abdominal surgery	RA, 50 or 70 mg kg^−1^, 3 mL poured over the lesion site.	[90]
Post-surgical adhesion	Sprague Dawley rats	Midline laparotomy incision	PGAcys hydrogel; PGAcys/RA hydrogel; hyaluronate/carboxymethylcellulose.	[91]
Parotid gland injury	Sprague Dawley rats	X-ray irradiation	RA, 30, 60, or 120 mg kg^−1^, i.g.; amifostine, 250 mg kg^−1^, i.p.	[92]
Skin wound	Wistar rats	Full-thickness skin wound	10% RA cream; 5% dexpanthenol cream, applied on the wound.	[93]
Skin wound	Sprague Dawley rats	Full-thickness skin wound	RA-grafted hydrogel, 50 μL, placed on the wound.	[94]

**Table 2:** Composition of herbal prescriptions and familial information of the plant species with antifibrotic effects.

Herbal Prescription or Plant Name	Plants of Lamiaceae	Plants of Other Families	Literature
Yang-Gan-Wan		*Angelica sinensis* (Oliv.) Diels (Apiaceae); *Plantago asiatica* L. (Plantago depressa Willd.) (Plantaginaceae); *Paeonia lactiflora* Pall. (Paeoniaceae); *Saposhnikovia divaricate* (Turcz.) Schischk. (Apiaceae); *Prinsepia utilis* Royle (Rosaceae); *Rehmanniae preparata* (Orobanchaceae); *Ligusticum scoticum* L. (Apiaceae); *Citrus aurantium* L. (Rutaceae)	[65,118]
Fuzheng Huayu recipe	*Salvia miltiorrhiza* Bunge	*Cordyceps militaris* (L.) Fr. (Cordycipitaceae); *Prunus persica* (L.) Batsch (Rosaceae); *Gynostemma pentaphyllum* (Thunb.) Makino (Cucurbitaceae); *Pinus massoniana* Lamb. (Pinaceae); *Schisandrae Chinensis* (Turcz.) Baill. (Magnoliaceae)	[101,102]
Wen-pi-tang-Hab-Wu-ling-san	*Salvia miltiorrhiza* Bunge; *Perilla frutescens* (L.) Britton	*Codonopsis pilosula* Franch. (Campanulaceae); *Pinellia ternata* (Thunb.) Makino (Araceae); *Coptis chinensis* Franch. (Ranunculaceae); *Epimedium koreanum* Nakai (Berberidaceae); *Rheum palmatum* L. (Polygonaceae); *Glycyrrhiza uralensis* Fisch. ex DC. (Fabaceae); *Artemisia capillaris* Thunb. (Asteraceae); *Alisma plantago-aquatica* var. *orientale* Samuels (Alismataceae); *Wolfiporia extensa* (Peck) Wolfiporia extensa (Peck) Ginns (*Poria cocos* (Schw.) Wolf (Polyporaceae); *Atractylodes macrocephala* Koidz. (Asteraceae); *Polyporus umbellatus* (Pers.) Fr. (Polyporaceae); *Cinnamomum cassia* (L.) J.Presl (Lauraceae)	[71,72,73]
Guanxinning injection	*Salvia miltiorrhiza* Bunge	*Ligusticum chuanxiong* Hort. (Apiaceae)	[78]
Ground ivy (gill-over-the-ground)	*Glechoma hederacea* L.		[66]
Lemon balm	*Melissa officinalis* L.		[69]
Xiang-Ru (Vietnamese balm)	*Elsholtzia ciliata* (Thunb.) Hyl.		[74]
Ze-Lan (hirsute shiny bugleweed)	*Lycopus lucidus* Turcz. ex Benth.		[77]
Basil	*Ocimum basilicum* L.		[79]
Xia-Ku-Cao (self-heal)	*Prunella vulgaris* L.		[80]
Rosemary	*Salvia rosmarinus* Spenn (*Rosmarinus officinalis* L.)		[85]
Danshen (redroot sage)	*Salvia miltiorrhiza* Bunge		[88,101]

## Data Availability

Not applicable.

## Abbreviations

ACC	Acetyl-CoA carboxylase
ACE	Angiotensin-converting enzyme
AGEs	Advanced glycation end-products
ALT	Alanine aminotransferase
AMPK	5′ AMP-activated protein kinase
Akt	Protein kinase B (PKB)
AP-1	Activator protein 1
Apaf-1	Apoptotic protease activating factor-1
ARE	Antioxidant response element
AST	Aspartate aminotransferase
AT1R	Angiotensin type 1 receptor
Bad	Bcl-2-associated death protein
BALF	Bronchoalveolar lavage fluid
Bcl 2	B cell lymphoma/leukemia 2
BDL	Bile duct ligation
BUN	Blood urea nitrogen
CAMK	Calcium/calmodulin-dependent protein kinase
COL	Collagen
CTGF	Connective tissue growth factor
DKK1	Dickkopf-1
ECM	Extracellular matrix
EMT	Epithelial–mesenchymal transition
ERK	Extracellular signal-regulated kinase
FAS	Fatty acid synthase
FZHY	Fuzheng Huayu recipe
G6PDH	Glucose-6-phosphate dehydrogenase
GCLc	Catalytic subunits of glutamate cysteine ligase
GPX	Glutathione peroxidase
GR	Glutathione reductase
GSH	Glutathione
GST	Glutathione S-transferase
HO	Heme oxygenase
HSCs	Hepatic stellate cells
HSF	Heat shock factor
HSPs	Heat shock proteins
IFN-γ	Interferon gamma
Ig	Immunoglobulin
IKK	IκB kinase
IL	Interleukin
JNK	c-Jun N-terminal kinase
KEAP	Kelch-like ECH-associated protein
LAD	Left anterior descending coronary artery
LPS	Lipopolysaccharide
MAPK	Mitogen-activated protein kinase
MCD	Methionine- and choline-deficient
MDA	Malondialdehyde
MDCKs	Madin–Darby canine kidney cells
MI	Myocardial infarction
MMP	Matrix metalloproteinase
NF-κB	Nuclear factor kappa B
NOS	Nitric oxide synthase
NOX	NADPH oxidase
NRF2	Nuclear factor erythroid 2-related factor 2
PCLS	Precision-cut liver slices
PCOL	Procollagen
PDGF	Platelet-derived growth factor
PDGFR	PDGF receptor
PGC	PPARγ coactivator
PI3K	Phosphoinositide 3-kinase
PKC	Protein kinase C
PLA	Phospholipase A
PLC	Phospholipase C
PPAR	Peroxisomal proliferator-activated receptor
RA	Rosmarinic acid
ROS	Reactive oxygen species
RXR	Retinoid X receptor
SD	Sprague Dawley
shRNA	Short hairpin RNA
SMA	Smooth muscle actin
SMAD	Small mothers against decapentaplegic
SOD	Superoxide dismutase
SREBP	Sterol regulatory element-binding protein
STAT	Signal transducers and activators of transcription
TGF	Transforming growth factor
TGF-βR	TGF-β receptor
TIMP	Tissue inhibitor of metalloproteinase
TLR	Toll-like receptor
TNF	Tumor necrosis factor
TNFR	TNF receptor
TrCP	Transducin repeat-containing protein
UUO	Unilateral ureteral obstruction
WHW	Wen-pi-tang-Hab-Wu-ling-san
